# Identifying lower limb problems and the types of safety footwear worn in the Australian wine industry: a cross-sectional survey

**DOI:** 10.1186/s13047-021-00495-3

**Published:** 2021-11-29

**Authors:** Alexander Willem Copper, Rolf Scharfbillig, Thuy Phuong Nguyen, Cassandra Collins

**Affiliations:** 1 Department of Wine Science and Waite Research Institute, The University of Adelaide. PMB 1, Glen Osmond, South Australia 5064 Australia; 2 School of Allied Health and Human Performance, University of South Australia, Adelaide, South Australia 5001 Australia; 3 Menzies Institute for Medical Research, College of Health and Medicine, University of Tasmania, 17 Liverpool St, Hobart, Tasmania Australia

**Keywords:** Safety footwear, Elastic sided safety boots, Lower limb, Occupational health, Wine industry

## Abstract

**Background:**

The Australian wine industry is a valuable part of the wider Australian economy worth approximately A$45 billion annually and employs 163,790 people either full time or part time. Australian agricultural industries are amongst the nation’s most dangerous workplaces with joint, ligament, muscle and tendon injuries being commonplace along with wounds, lacerations and musculoskeletal diseases. It is therefore important to try and minimise the risk of injuries to workers. The aims of this study were to (1) identify whether lower limb problems occur in the Australian wine industry and (2) identify the types of safety footwear worn.

**Methods:**

Participants were recruited from the Australian wine industry. The study was a cross-sectional anonymous survey of 82 questions with *n* = 207 respondents. Questions related to job role performed, types of lower limb problems experienced, level of pain, restriction of activities, types of footwear worn, general health and physical health.

**Results:**

The main working roles were winery (73.4%), vineyard (52.2%), laboratory (39.6%), cellar door (32.4%) and office (8.2%), with 63.3% of participants working in more than one role. Lower back pain was the most commonly reported problem at 56% followed by foot pain (36.7%), knee pain (24.6%), leg pain (21.3%), ankle pain (17.9%), hip pain (15.5%), toe pain (13%) and heel pain (11.1%). The most popular footwear used by participants were elastic sided safety boots, followed by high cut lace up safety boots with side zip. Overall, although the pain experienced was moderate, it did not impact the workers ability to perform their duties and the majority self-reported as being in very good general and physical health.

**Conclusion:**

To date no data have been published on the types of lower limb problems or the types of safety footwear worn in the Australian wine industry. This study is the first to demonstrate that elastic sided safety boots were the most popular amongst respondents and that lower limb problems occur with workers. Therefore, further research into the safety footwear used in the Australian wine industry is needed to better support workers health while working in their varied roles and conditions.

**Supplementary Information:**

The online version contains supplementary material available at 10.1186/s13047-021-00495-3.

## Background

The Australian wine industry is a valuable part of the wider Australian economy, worth approximately A$45 billion annually and employing 163,790 people either full time or part time [1]. Therefore, the wine industry and the health and safety of its workers are an important part of Australian society and the economy. The industry is also somewhat unique, in that most businesses are small to medium in size and consist of multiple workplace environments within the one entity [2]. In 2020 there were 2361 wineries and 6251 grape growers in Australia. Approximately 64% of producers are considered small to medium and process less than 50 t of grapes per year, 20% process 50–499 t and 16% process more than 500 t [2].

Wine business owners and employees may work in various combinations of roles across the business, particularly in smaller, family run enterprises. Small wineries are comprised of a primary industry (grape growing), a secondary process (wine production) and tertiary activities such as restaurants and cellar door sales [3]. Grape growing activities can include operating heavy machinery as well as driving tractors and harvesters in the vineyard. Winery activities can include operating forklifts/pumps/crushers/conveyor belts and bottling machines, along with analysing juice and wine samples in the laboratory plus general office work. Sales and hospitality work in the cellar door can often include food service. Most of these activities involve wearing protective safety footwear and standing for long periods. This is particularly the case during the vintage/harvest season, when the weather is very hot and shifts are longer than usual due to the time constraints involved in harvesting and processing grapes at the optimal time.

To date no data have been published on the types of injuries experienced in the Australian wine industry and the cost of injury to the industry. However, the South Australian government, which is the largest wine production area in Australia and accounts for 52% of the national output, publishes data on workplace injuries across several industries [2, 4]. The majority of injuries reported by the South Australian government in 2020 were for technicians/trade workers (27.9%), labourers (26.6%) and machinery operators/drivers (16%). The injuries were predominantly upper limbs (38.5%), lower limbs (18.9%) and trunk/back (17.9%). The nature of the injuries were mainly traumatic joint/ligament/muscle/tendon injuries (37%), wounds/lacerations (29.1%) and musculoskeletal diseases (15.2%). The main mechanism for these injuries were body stresses (34.5%), being hit by an object (19.4%) and falls/trips/slips (17.3%) [4]. In 2012–13, work-related injury and disease cost the Australian economy A$61.8 billion, representing 4.1% of the gross domestic product [5].

Injuries among vineyard workers appear to be common. A study in France found that vineyard workers were likely to experience musculoskeletal pain. They reported upper limb pain (31.2%), neck/shoulder (28.9%), lower limb (25%) and back pain (55%) prevalence during grapevine pruning and grape harvesting [6]. Similar results have been seen with vineyard workers in the United States [7–9], Italy [10] and Argentina [11], but no specific data exists for Australian vineyard workers or winery workers in general.

Whilst there is limited data available for the wine industry, much can be gleaned from other agricultural industries that are reportedly some of the most dangerous workplaces [12]. For example, manual harvesting is prevalent in many agriculture industries and is the largest contributor to work-related musculoskeletal disorders [12]. The Australian aquaculture industry reports that 37.3% of injuries are body stressing events and that lower limb injuries account for 20.3% of all injuries [13]. The majority of these industries involve spending prolonged hours standing and it has been demonstrated that this can increase the risk of musculoskeletal disorders such as lower back, lower extremity and foot disorders [14].

Work environment flooring and footwear have also been demonstrated as risk factors [15], considering the variable nature of wine industry work environments, this is an area that warrants further investigation. Safety boots are compulsory in many occupations to protect the feet of workers from external stimuli, particularly in harsh environments [16]. The unique environmental conditions and tasks in different occupations necessitates a variety of boot designs to match each workers occupational requirements [17]. Unfortunately, safety boots are often designed more for safety at the expense of functionality and comfort [17].

Further risk factors for agricultural and horticulture workplace injuries include working full time, being the owner/operator, medication use, prior injury, poor health, stress/depression, poor hearing [18], heat stress [19] and inadequate sleep due to shift work [20, 21].

When these risk factors and the nature of wine industry work are taken into consideration, especially during the busy vintage/harvest period, it can be seen that wine industry work environments pose a potential risk to injury. Therefore, the aims of this study were to (1) identify whether lower limb problems occur in the Australian wine industry and (2) identify the types of safety footwear used.

## Methods

### Survey design and testing

The study was cross-sectional with the design based on previous validated surveys, questionnaires and studies that investigated foot health [22], musculoskeletal discomfort [23, 24] and the footwear needs of workers [17, 25]. Content validity was considered via discussions with podiatry, physiotherapy, occupational injury and wine industry representatives and the survey questions were modified so as to be appropriate for the wine industry and capture the relevant areas of concern. Reliability was established by trialling the survey on 10 participants who completed the survey anonymously. Four weeks later the same 10 participants completed the survey a second time to test for repeatability and to ensure the questions were well understood.

The final survey consisted of 82 closed-ended questions (Likert scale and choose all that apply), that were divided in to nine sections including job role, lower limb problems at work, treatment sought, severity of pain, limitations caused by lower limb problems, types of footwear worn at work, footwear fit & comfort, general health and physical health (see supplementary information, Additional file 1 for survey).

Participants were recruited by several methods: writing to wine and grape industry bodies throughout Australia requesting surveys be distributed to members, supplying surveys to the work health and safety manager of the largest corporate wine company in Australia, emailing wine industry workers at the University of Adelaide and online via wine industry social media groups. Participants self-selected to complete the anonymous survey (*n* = 207) and the survey was open for 2 weeks after the Australian vintage/grape harvest period in May 2021.

For large populations, it is recommended that surveys have a sample size of 188 for 90% confidence level and 267 for 95% confidence level [26]. Therefore, for *n* = 207 and confidence level of 95%, the confidence interval was calculated as 6.8% [27].

Human Research Ethics Committee approval for the survey was given by the University of Adelaide (H-2020-267). An implied consent statement was placed at the beginning of the survey indicating that continuation with the questionnaire implied the participants consent.

### Survey items

#### Job role

Participants were asked what job roles they performed in the Australian wine industry in the last 12 months. The question was a choose all that apply, closed-ended question with the option that workers could have several different roles within their workplace.

#### Lower limb problems at work

Lower limb aches, pain and injuries were assessed by asking participants if they had experienced any problems in different body areas in the last 12 months. If the participant had no lower limb problems, they were directed to the footwear section of the survey.

#### Treatment sought

Those participants that had experienced lower limb problems at work were asked if they had been hospitalised due to these problems or if they had sought any treatment.

#### Severity of pain

A Likert scale was used for participants to rate their pain (1 ‘low’ to 5 ‘severe’). Likewise, a Likert scale (1 ‘never’ to 5 ‘always’) was used to determine; the frequency of pain, if pain limited work duties possible and any difficulties in completing work activities.

#### Limitations caused by lower limb problems

The final question regarding lower limb problems was related to how these problems affected daily activity in general, not only in work situations. A Likert scale (1 ‘not at all’ to 5 ‘always’) was used to rate any limitations.

#### Types of footwear worn

Participants were asked a closed-ended question relating to the style of footwear they most often used at work. A choose all that apply format was used and they were also asked if they use any additional support or cushioning in these shoes. The types of footwear were separated in to two groups: safety or non-safety.

#### Footwear fit and comfort

Participants were asked to rate their impression of their footwear’s fit and comfort using a Likert scale (1 ‘strongly disagree’ to 5 ‘strongly agree’) over a series of 19 questions.

#### General health

Participants were asked to rate their general health with a Likert scale (1 ‘poor’ to 5 ‘excellent’) and answer nine questions relating to their general wellbeing using a Likert scale (1 ‘definitely false’ to 5- ‘definitely true’).

#### Physical health

The final five questions related to general physical health and participants used a Likert scale (1 ‘definitely false’ to 5 ‘definitely true’) to rate their responses.

### Statistical analysis

Data sorting and preparation were conducted with Microsoft Excel 2010, the closed-ended questions and Likert scale questions were counted to determine frequencies. Descriptive statistics and one-way ANOVA for the reliability trial were performed using the statistical package XLSTAT (version 2019.4.2, Addinsoft SARL, Paris, France).

Correspondence analysis and Polychoric correlation factor analysis was performed on the binary data relating to job role, area of lower limb problem and type of footwear worn using the statistical package XLSTAT (version 2019.4.2, Addinsoft SARL, Paris, France). Polychoric correlation factor analysis is the preferred method for studying the construct validity of exploratory and confirmatory data when using Likert scales and binary questionnaires [28]. Correspondence analysis is a statistical technique recommended for multivariate analysis of contingency tables. This analysis is used to graphically display the association in two-way categorical data [29].

## Results

### Reliability trial

Due to the anonymous nature of the survey, no respondent identification codes were used. Consequently, the use of a t-test for reliability was not possible. One-way ANOVA analysis was therefore used for the repeated trial survey to determine any differences between the response means for each question after a 4 week interval. There were no significant differences between the response means for each question, with *p*-values for each question ranging from 0.15 to 1.0.

### Job role

The main working roles reported by the participants were winery (73.4%), vineyard (52.2%), laboratory (39.6%), cellar door (32.4%) and office (8.2%). Interestingly 63.3% of participants worked in more than one role, highlighting the multifaceted nature of wine industry work.

### Lower limb problems

Lower back pain was the most commonly reported problem at 56% followed by foot pain (36.7%), knee pain (24.6%), leg pain (21.3%), ankle pain (17.9%), hip pain (15.5%), toe pain (13%) and heel pain (11.1%). Respondents who reported no problems at work were directed to the footwear, general and physical health question section.

### Treatment sought

The most common practitioner survey participants sought out for treatment were physiotherapists (36.2%). Surprisingly, the same frequency of participants sought no treatment for their problems. Other practitioners consulted were general practitioners/medics (18.8%), podiatrists (18.4%), massage therapists (18.4%), chiropractors (16.4%), osteopaths (6.3%), surgeons (5.8%) and 14% had been hospitalised because of their problems.

### Severity of pain

For participants that reported lower limb problems whilst working, pain was is in the ‘mild’ to ‘moderate’ range. How often this pain was experienced was in the ‘occasionally’ to ‘very often’ range. Whilst results for whether the pain limited work duties or caused difficulties performing work activities were in the ‘occasionally’ to ‘many times’ range (Table 1).

**Table 1:** Responses to questions relating to severity of pain at work

	Question	Mean	SD	F1%	F2%	F3%	F4%	F5%
(a)	Rate the level of pain you experienced in the last 12 months.	3.47	0.96	4.9	8.6	30.8	45.8	9.9
(b)	How often did you experience this pain?	2.94	1.01	0	43.8	20.9	27.7	7.6
	Were you limited in the duties you could do at work?	2.12	0.99	27.7	45.2	14.8	11.1	1.2
	Has it caused you to have difficulties in your work activities?	2.26	0.89	16.6	52.5	20.4	9.3	1.2

Response range and definition: (a) 1-low, 2-very mild, 3-mild, 4-moderate, 5-severe. (b) 1-never, 2-occasionally, 3-many times, 4-very often, 5-always. F1, F2, F3, F4, F5- Frequency (%) of response 1, 2, 3, 4, 5. *Mean* response mean, *SD* standard deviation

### Limitations caused by lower limb problems

Overall the pain experienced by participants did not limit their ability to perform several activities (Table 2). Most activities were between ‘not at all’ and ‘a little’. Only vigorous activities, bending and climb a hill were in the ‘a little’ to ‘moderate’ range.

**Table 2:** Response to question; during a typical day how much does this pain interfere with the following activities

Question	Mean	SD	F1%	F2%	F3%	F4%	F5%
Vigorous Activities	2.65	1.19	14.8	39.5	21.6	14.2	9.9
Moderate Activities	1.98	0.91	34.4	41.3	16.2	8.1	0
Lift small objects such as shopping bags	1.59	0.86	59.4	26.9	10	2.5	1.2
Climb a hill	2.06	1.15	39.4	31.3	19.4	3.6	6.3
Walk up a flight of stairs	1.84	1.1	50.6	28.8	11.8	3.7	5.1
Bend	2.20	1.13	31.8	36.3	16.3	11.9	3.7
Walk 1 km	1.89	0.99	48.1	24.4	20	7.5	0
Walk 100 m	1.51	0.78	64.4	23.1	10	2.5	0
Shower yourself	1.36	0.68	71.9	24.4	2.5	0	1.2

Response range and definition: 1-not at all, 2-a little, 3-moderately, 4-very often, 5-always. *Mean* response mean, *SD* standard deviation. F1, F2, F3, F4, F5- Frequency (%) of response 1, 2, 3, 4, 5

### Types of footwear worn

The most popular footwear used by participants were elastic sided safety boots, followed by high cut lace up safety boots with side zip (Table 3). Additional support or cushioning in shoes was reportedly used by respondents, however no details on the types of support or cushioning were recorded. That is, whether it was a custom-made foot orthosis or an off the shelf insole.

**Table 3:** Response to types of footwear most often worn at work

Boot Style	Frequency %
Elastic sided safety boots	46.4
High cut lace up safety boots with side zip	25.1
Sports shoe	15.0
High cut safety boots with laces	14.0
Elastic sided boots (not safety)	9.7
Rubber/Wellington/Gum boots	9.7
Dress shoe	9.7
Low-Mid cut safety shoes with laces	7.7
Use additional support or cushioning in your shoes	31.4

### Footwear fit and comfort

The majority of participants reported that their work footwear was comfortable (Table 4), however more than a third of respondents reported that their shoes made their feet ache at work and that their shoes made their feet hurt after work. More than half of the respondents reported their boots as being hot and heavy. Some respondents reported difficulty in; finding shoes that did not hurt their feet, finding shoes to fit their feet and that their shoes were heavy. Overall, respondents thought; their shoes had good grip, their shoes were easy to take off and put on, their shoes fit well, had good ankle support, were durable, good value, felt safe and protected when wearing their footwear and they like the style.

**Table 4:** Responses to questions regarding the fit and comfort of the footwear worn

Question	Mean	SD	Disagree (%)	Agree (%)
It is hard to find shoes that do not hurt my feet.	2.87	1.39	49.3	37.2
I have difficulty in finding shoes that fit my feet.	2.83	1.30	49.2	38.1
I am limited in the number of shoes that I can wear.	2.96	1.33	46.4	46.9
My shoes are comfortable.	3.59	1.00	18.4	63.8
My shoes have good arch support.	3.16	1.05	29.0	38.2
My shoes are cushioned.	3.47	0.99	18.4	58.5
My shoes make my feet ache when I am at work.	2.96	1.26	40.1	33.8
My shoes make my feet hurt after work.	3.01	1.25	41.5	41.1
My shoes have good grip.	4.10	0.74	3.4	85.5
My shoes make my feet feel hot.	3.41	1.03	23.2	56.5
My shoes are durable.	3.63	0.95	15.0	67.6
My shoes are easy to put on and take off.	4.01	0.96	10.6	79.2
My shoes fit well.	3.82	0.98	14.0	73.9
My shoes are heavy	3.55	1.02	17.9	58.9
My shoes are good value for money.	3.48	1.02	12.1	50.7
I like the style of my shoes.	3.68	1.00	12.6	63.8
I feel safe and protected when wearing my shoes.	4.02	0.76	2.9	79.7
My shoes provide good ankle support.	3.37	1.08	28.5	54.6
My shoes are waterproof.	2.70	1.14	51.2	30.9

Response range: 1-strongly disagree, 2-disagree, 3-neither agree nor disagree, 4-agree, 5-strongly agree. *Mean* response mean, *SD* standard deviation

### General health

The majority of participants’ general health was reported as being ‘very good’ to ‘excellent’ on the Likert scale, while very few reported their health as ‘fair’ to ‘poor’ (Table 5).

**Table 5:** Response to general health and wellbeing questions

	Question	Mean	SD	% 1–2	% 4–5
(a)	How would you describe your general health?	3.81	0.67	1.5	69.1
(b)	I seem to get sick a lot easier than most people	1.68	0.85	66.7	4.3
	I am as healthy as anybody I know	3.78	0.97	12.1	73.4
	I expect my health to get worse	2.61	1.14	44.9	23.7
	My health is excellent	3.67	1.04	19.3	71.5
	I feel full of life	3.56	1.04	23.2	61.8
	I feel tired	3.24	1.25	36.7	54.1
	I feel calm	3.42	1.04	27.1	59.9
	I feel happy	3.80	0.95	15.5	74.4
	I feel depressed	2.29	1.16	65.2	21.7

Response range: (a): 1-poor, 2-fair, 3-average, 4-very good, 5-excellent. (b): 1-definitely false, 2-mostly false, 3-don’t know, 4-mostly true, 5-definitely true. *Mean* response mean, *SD* standard deviation

Overall, the participants agreed with statements describing themselves as healthy and happy. They were as ‘healthy as anyone I know’, full of life, calm and happy. However, 54.1% agreed with the statement ‘I feel tired’ and 21.7% agreed with the statement ‘I feel depressed’.

### Physical health

The participants reported on the whole that their overall physical health did not impede the types of activities they were able to achieve during work. Mean values for all questions relating to physical health ranged from ‘definitely false’ to ‘mostly false’ on the Likert scale (Table 6).

**Table 6:** Responses to the questions; during the past 12 months, how much of the time have you had any of the following problems with your work or other activities as a result of your physical health?

Question	Mean	SD	False (%)	True (%)
Reduced the amount of time you spent on work or other activities.	1.68	0.80	85.9	1.9
Accomplished less than you would like.	1.85	0.85	77.3	3.3
Were limited in the kind of work or other activities.	1.87	0.89	78.2	3.8
Took extra time performing work or other activities.	1.89	0.83	79.2	2.9
Interfered with normal social activities with family and friends.	1.53	0.76	86.1	0.9

Response range and definition: 1-definitely false, 2-mostly false, 3-don’t know, 4-mostly true, 5-definitely true. *Mean* response mean, *SD* standard deviation

### Correlation analysis

Fourteen correlations were identified but only two correlations relating to job role were seen (Table 7). That is, working at the cellar door and wearing sports shoes and a negative correlation for working in the office and wearing low-mid cut safety shoes as well as toe pain. The negative correlation indicates that a person working in an office is less likely to wear low-mid cut safety shoes and less likely to have toe pain. Wearing gum boots and knee pain had a moderate correlation while wearing low-mid cut safety shoes was negatively correlated with hip pain. Wearing dress shoes was negatively correlated with heel pain and ankle pain. Finally, elastic sided boots (not safety) were negatively correlated with hip, ankle and leg pain.

**Table 7:** Summary of polychoric correlation matrix of variables job role, footwear worn and lower limb problem (full matrix in Additional file 2)

Variables	Correlation
Foot pain x Toe pain	0.8
Heel pain x Toe pain	0.8
Cellar Door x Sports shoes	0.6
Leg pain x Ankle pain	0.5
Leg pain x Foot pain	0.5
Knee pain x Gum boots	0.5
Toe pain x Office	−0.9
Hip pain x Low-Mid cut safety shoes	−0.9
Hip pain x Elastic sided boots	−0.9
Heel pain x Dress shoes	−0.9
Ankle pain x Elastic sided boots	−0.9
Ankle pain x Dress shoe	−0.9
Leg pain x Elastic sided boots	−0.9
Office x Low-Mid cut safety shoes	−0.8

Moderate correlation 0.5 to 0.7, high correlation 0.7–1.0.

Negative correlation indicates inverse relationship of variables

Correspondence analysis showed significant association between footwear worn and lower limb problems. In particular elastic sided safety boots were associated with hip, ankle, leg, lower back and foot pain (Table 8). High cut lace up safety boots with side zip were associated with heel, foot, toe and lower back pain. High cut lace up safety boots were associated with leg and ankle pain. Gum boots were associated with knee and ankle pain. These associations between footwear and site of problem, however, do not imply causation.

**Table 8:** Correspondence analysis of relationship between the footwear worn and the lower limb problem

	ElastBS	LaceZip	HSBL	LMSBL	ElasB	Sport	Gum	Dress
Lower back	32%	20%	12%	3%	6%	10%	7%	11%
Hip	45%	18%	5%	0%	0%	14%	9%	9%
Leg	39%	14%	18%	7%	0%	12%	7%	4%
Knee	31%	17%	10%	3%	4%	15%	15%	5%
Ankle	40%	7%	18%	4%	0%	16%	15%	0%
Feet	32%	24%	8%	9%	6%	9%	6%	7%
Heel	20%	31%	6%	6%	14%	6%	17%	0%
Toe	30%	23%	5%	14%	5%	9%	9%	5%

χ^2^ = 82.9, *p-*value 0.002, α = 0.05.

*ElastBS* elastic sided safety boots, *LaceZip* high cut lace up safety boots with side zip, *HSBL* high cut lace up safety boots, *LMSBL* low/mid cut lace up safety shoes, *ElasB* elastic sided boots, Sport- sports shoe, *Gum* gum/wellington boots, *Dress* formal or dress shoe

## Discussion

To the best of our knowledge, this preliminary study is the first of its kind to identify whether lower limb problems occur in the Australian wine industry and what types of safety footwear are worn. It has also taken a snapshot of how these problems affect workers and how workers perceive their footwear. The survey was not comprehensive however, it has provided valuable information for developing further research into the future. For example, the nature of the work involved in each role was not explored in great detail, that is, how much time workers spent standing, walking or sitting in each role. Previous research has reported that an estimated 50% of the working population experience musculoskeletal disorders due to prolonged standing and that standing is implicated in lower back, lower limb and foot pain [30, 31].

Lower back pain was the most commonly reported problem at 56% followed by foot pain (36.7%), If foot, toe and heel pain are combined to total foot pain, 60.8% of respondents experienced some type of foot pain. Only 19.3% of respondents reported no lower limb problems, which is a common limitation of such self-selected surveys [18]. The results are similar to those reported for miners and their work boots, that is, lower back pain (44.5%), foot pain (42.3%), knee pain (21.5%) and ankle pain (24.9%) [18].

The majority of lower limb pain experienced by participants was in the ‘mild’ to ‘moderate’ range (Table 1) and it was experienced ‘occasionally’ to ‘many times’, it did not however have a large impact on the ability for workers to complete their daily activities. Only vigorous activities, bending and climb a hill were in the ‘a little’ to ‘moderate’ range (Table 2). This could explain the unwillingness of participants to seek treatment for lower limb problems, as they may have felt that it was a ‘normal’ part of their work. Overall, physical health wasn’t reported as being a concern, as was seen in Table 6, with the majority of respondents disagreeing with the statements about problems with work or other activities as a result of their physical health.

The majority of participants’ general health was reported as being ‘very good’ to ‘excellent’ with very few reporting that their health was ‘fair’ to ‘poor’ (Table 5). However, 54.1% agreed with the statement ‘I feel tired’ and 21.7% agreed with the statement ‘I feel depressed’. This figure for depression is similar to that reported by the Australian Bureau of Statistics [32] where 20.1% of Australians reported themselves as having an anxiety-related condition and depression or feelings of depression. While this survey was conducted during the SARS-CoV-2 pandemic, which may have impacted the mental health of the respondents, at the time of the survey no Australian states were under government sanctioned stay at home orders, but international travel restrictions were in place.

Respondents reported using additional support or cushioning in their shoes, however no details on the types of support or cushioning were recorded. That is, whether it was a custom-made foot orthosis or an off the shelf insole. Dobson et al. [17] reported that in the mining cohort they studied, only 6.7% of respondents wore health professional prescribed orthoses. Flat insoles and contoured foot orthoses have been shown to increase plantar pressures at the midfoot, reduce plantar pressures at the rearfoot, and provide small reductions in tibial accelerations when used in high cut, lace up, boots [33]. However, no differences in ‘boot comfort’ between the no insole, flat insole and contoured foot orthosis groups were identified in this previous study [33]. Therefore, no conclusions can be made as to whether the use of additional support or cushioning had an effect on respondents’ comfort in this preliminary survey.

Elastic sided safety boots were the most popular, followed by the high cut lace up safety boots with side zip. Even if high cut lace up safety boots are considered analogous to the high cut lace up safety boots with side zip, elastic sided safety boots are still the most popular. Also, workers that wear elastic sided safety boots are more likely to experience lower limb problems such as foot and lower back pain, however no link between footwear and lower limb pain can be inferred from this study. Safety footwear was also reported as being hot and heavy in this study. Heavy footwear has been associated with increased energy expenditure by workers wearing safety footwear [34]. Dobson et al. [17] report that 62.3% of miners believed that their foot and ankle pain was related to their work boots. One explanation for this is that miners may be wearing boots that are longer than their feet, possibly because boots in their correct length are too narrow [35]. Dobson et al. [36] concluded that traditional fitting methods based on foot length were insufficient when fitting miners. Grau and Barisch-Fritz [37] concur and state that foot width and girth measures are different in static and dynamic loading situations and must be considered when manufacturing and fitting safety footwear to aid in supporting workers health. Buldt and Menz [38] state that between 63 and 72% of the general population are wearing inappropriately sized footwear based on length and width measurements, and that incorrect footwear fitting is significantly associated with foot pain. In this study however, 73.9% agreed with the statement that their footwear fit well.

Many studies have explored the relationship between safety footwear and injuries with the majority focusing on; high cut lace up safety boots, military boots, gum boots, sports shoes [16] and surgical clogs [31]. These studies have identified many relationships, for example, gum boots are associated more with knee and heel pain while high cut lace up safety boots were associated with more leg and ankle pain [39]. Gum boots are associated with more force and contact area in the heel compared to the high cut lace up safety boots [40]. High cut lace up safety boots with varying sole and shaft stiffness are associated with effects on lower limb muscle activity, ankle motion [41] and plantar pressures [42]. High cut lace up safety boots also have an impact on postural control [43] and postural stability under workload [44]. High cut lace up military boots while carrying a workload also have an effect on postural stability and heel contact during slip events [45, 46].

To date no data exists on the effect of elastic sided safety boots in any industry. There is also no published data on how safety footwear is supplied to workers and protocols for when it is replaced in the wine industry. Recent research into shoe tread (sole) wear and wear measurement has highlighted the need for understanding the mechanism for shoe tread wear and individualised shoe replacement recommendations to prevent injury caused by the decline in traction of worn shoes [47–49].

The popularity of elastic sided safety boots in the Australian wine industry is a unique phenomenon and its use therefore may be more due to tradition. Elastic sided boots were developed in the early 1900s to withstand the harsh, unforgiving environment of the Australian outback by providing a boot that was comfortable, rugged and able to cope with both hot/dry and cold/wet seasons [50]. They became popular heavy-duty footwear for farming, forestry, mining, and industrial uses [50]. Another reason for the popularity of elastic sided safety boots may be the nature of the work in the Australian wine industry. As has been highlighted, many wine businesses require workers to perform varying jobs over different sites and conditions, this often involves a quick change of suitable footwear, 79.2% of respondents agreed with the statement that their shoes are easy to put on and take off, which could also help to explain the popularity of elastic sided safety boots.

Boot design features have been shown to have an influence on the lower limbs depending on the task being performed and the supporting surface [16]. Therefore, occupational specific testing of footwear effects should occur in the Australian wine industry in order to try and accommodate for individual workplace environments.

## Conclusion

This study has shown that lower limb problems occur in the Australian wine industry and that even if a problem is present workers often do not seek treatment or let the problem interfere with their work activities. This may be a function of the vintage/harvest season, when harvesting and processing grapes at their optimum condition places time constraints on workers. The study also demonstrated that elastic sided safety boots were the most popular amongst respondents.

These factors highlight the need for further research into the footwear used in the Australia wine industry to better support workers health while working in varied roles and conditions. A comparison of different footwear in different environments could take place as well as exploring the time taken for footwear to deteriorate in these environments. The optimum length of efficacy of the footwear could also be assessed to ensure footwear is replaced at appropriate times and not used when worn excessively. Future research is warranted to determine any barriers and facilitators regarding boot choice in the wine industry, especially when the multi-faceted work environments and the appropriate footwear for specific roles are taken into consideration.

As is the case with all surveys, there are limitations to this study and the accuracy of self-reported measures. This may have caused some selection bias, with participants who have experienced lower limb problems more likely to self-select to participate in the survey.

Recall bias can also be a problem, an attempt to minimise this risk was to set a 12 month limit on the survey, that is, participants were asked if they had worked in the Australian wine industry in the last 12 months. If they had not, they were directed to leave the survey. No demographic data on the participants were collected in this preliminary survey. Future research involving randomised control trials of specific footwear could include the collection of demographic data for more detailed analysis. Finally, it is not possible to conclude whether specific job roles had higher risks for lower limb problems with specific footwear. Correlations of these two factors does not imply any causation and requires further research to determine any relationship.

## Supplementary Information

**Additional file 1.** Copy of Survey

**Additional file 2.** Full polychoric correlation matrix of variables; job role, footwear worn and lower limb problem.

## Data Availability

The dataset used and analysed during this study is available from the corresponding author on reasonable request.

## Abbreviations

A$: Australian Dollars
ANOVA: Analysis of variance
ElastBS: Elastic sided safety boots
LaceZip: High cut lace up safety boots with side zip
HSBL: High cut lace up safety boots
LMSBL: Low/mid cut lace up safety shoes
ElasB: Elastic sided boots
Sport: Sports shoe
Gum: Gum/wellington boots
Dress: Formal or dress shoe
