# Supplementary material for: Identifying lower limb problems and the types of safety footwear worn in the Australian wine industry: a cross-sectional survey
Source: J Foot Ankle Res. 2021 Nov 29;14:58. doi: 10.1186/s13047-021-00495-3 (PMC8628050; doi:10.1186/s13047-021-00495-3)
Supplement: Supplementary file 1 — Additional file 1. Copy of Survey [file 13047_2021_495_MOESM1_ESM.pdf]

EXIT

THE UNIVERSITY of ADELAIDE

Incidence of lower limb injuries in the Australian wine industry 2021

Submission of your response is considered as your consent to participate and that you have read and understood the participant information.

1. Have you worked in the Australian wine industry in the last **12 months**? Choose all that apply.

- ☐ Winery
- ☐ Vineyard
- ☐ Cellar Door/Tasting Room
- ☐ Laboratory
- ☐ No (please exit the survey)
- ☐ Other (please specify)

2. How would you describe your **general health**?

- ○ Excellent

- ◯ Very good
- ◯ Average
- ◯ Fair
- ◯ Poor

3. In the past **12 months** have you experienced any pain, aches or injuries related to your work? Choose all that apply.

- ☐ Lower back
- ☐ Hip
- ☐ Leg
- ☐ Knee
- ☐ Ankle
- ☐ Feet
- ☐ Heel
- ☐ Toe
- ☐ Other (please specify)

- ☐ None of the above (go to question 9)

4. Have you ever seen a health professional because of the pain? Choose all that apply

- ☐ General practitioner (GP)

- [ ] Surgeon
- [ ] Podiatrist
- [ ] Physiotherapist
- [ ] Chiropractor
- [ ] Osteopath
- [ ] Massage therapist
- [ ] Other (please specify)

- [ ] None of the above

5. Have you ever been hospitalised because of the pain, aches or injuries?

- ○ Yes
- ○ No

6. Please rate the level of pain you experienced in the last 12 months.

Low	Very mild	Mild	Moderate	Severe	N/A
○	○	○	○	○	○

7. These questions relate to your level of pain you experienced in the last 12 months.

	Never	Occasionally	Many times	Very often	Always	N/A

	Never	Occasionally	Many times	Very often	Always	N/A
How often did you experience this pain?	○	○	○	○	○	○
Were you limited in the kind of work you could do because of your pain?	○	○	○	○	○	○
During the last 12 months has this pain caused you to have difficulties in your work activities?	○	○	○	○	○	○

8. During a **typical day** how much does the pain interfere with the following activities?

	Not at all	A little	Moderately	Very often	Always	N/A
Vigourous activity such as running, lifting heavy objects, participate in strenuous sport.	○	○	○	○	○	○

	Not at all	A little	Moderately	Very often	Always	N/A
Moderate activity such as cleaning the house, lifting a chair, participate in gentle sport.	○	○	○	○	○	○
Lifting or carrying bags of shopping.	○	○	○	○	○	○
Climbing a steep hill.	○	○	○	○	○	○
Climbing one flight of stairs.	○	○	○	○	○	○
Bending or kneeling.	○	○	○	○	○	○
Walking more than one kilometre.	○	○	○	○	○	○
Walking one hundred metres.	○	○	○	○	○	○
Showering or dressing yourself.	○	○	○	○	○	○

9. During the past **12 months**, how much of the time have you had any of the following problems with your work or other daily activities as a result of your **physical health?**

	Not at all	Slightly	Moderately	Most of the time	All of the time	N/A
Cut down on the amount of time you spent on work or other activities.	○	○	○	○	○	○
Accomplished less than you would like.	○	○	○	○	○	○
Were limited in the kind of work or other activities.	○	○	○	○	○	○
Had difficulty performing work or other activities (took extra time).	○	○	○	○	○	○
Interefered with your normal social activities with family, friends, neighbours or groups.	○	○	○	○	○	○

10. How true or false are the following statements about your **general health** to you?

	Definitely false	Mostly false	Don't know	Mostly true	Definitely true	N/A
I seem to get sick a lot easier than most people.	○	○	○	○	○	○

	Definitely false	Mostly false	Don't know	Mostly true	Definitely true	N/A
I am as healthy as anybody I know.	○	○	○	○	○	○
I expect my health to get worse.	○	○	○	○	○	○
My health is excellent.	○	○	○	○	○	○
I feel full of life.	○	○	○	○	○	○
I feel tired.	○	○	○	○	○	○
I feel calm and peaceful.	○	○	○	○	○	○
I feel happy.	○	○	○	○	○	○
I feel depressed.	○	○	○	○	○	○

11. What types of shoes do you wear most often at work? Choose all that apply.

- ☐ Elastic sided safety boot (no laces)
- ☐ Safety boot with side zip and laces
- ☐ Low cut/below ankle safety shoe (with laces)
- ☐ Mid cut/ankle height safety boot (with laces)
- ☐ High cut/above ankle safety boot (with laces)
- ☐ Slip on/safety shoe (no laces)

☐ Elastic sided boot (no safety toe protection)

☐ Sports shoe

☐ Fashion shoe

☐ Rubber boot/Wellington boot

☐ Other (please specify)

☐ None of the above

12. Do you use any additional cushioning or support in your work shoes?

☐ Yes

☐ No

13. The following questions concern the shoes you wear most often at work.

	Strongly disagree	Disagree	Neither agree or disagree	Agree	Strongly agree	N/A
It is hard to find shoes that do not hurt my feet.	○	○	○	○	○	○
I have difficulty in finding shoes that fit my feet.	○	○	○	○	○	○

	Strongly disagree	Disagree	Neither agree or disagree	Agree	Strongly agree	N/A
I am limited in the number of shoes that I can wear.	◯	◯	◯	◯	◯	◯
My shoes are comfortable.	◯	◯	◯	◯	◯	◯
My shoes have good arch support.	◯	◯	◯	◯	◯	◯
My shoes are cushioned.	◯	◯	◯	◯	◯	◯
My shoes make my feet ache when I am at work.	◯	◯	◯	◯	◯	◯
My shoes make my feet ache after work.	◯	◯	◯	◯	◯	◯
My shoes have good grip.	◯	◯	◯	◯	◯	◯
My shoes make my feet hot.	◯	◯	◯	◯	◯	◯
My shoes are durable (last a long time).	◯	◯	◯	◯	◯	◯
My shoes are easy to put on and take off.	◯	◯	◯	◯	◯	◯

	Strongly disagree	Disagree	Neither agree or disagree	Agree	Strongly agree	N/A
My shoes fit well.	○	○	○	○	○	○
My shoes are heavy.	○	○	○	○	○	○
My shoes are good value for money.	○	○	○	○	○	○
I like the style of my shoes.	○	○	○	○	○	○
I feel safe and protected when wearing my shoes.	○	○	○	○	○	○
My shoes provide good ankle support.	○	○	○	○	○	○
My shoes are water proof.	○	○	○	○	○	○

Thank you for taking part in this survey.

Done

Powered by
SurveyMonkey
See how easy it is to create a survey.

Privacy & Cookie Policy
