# Supplementary material for: Identifying lower limb problems and the types of safety footwear worn in the Australian wine industry: a cross-sectional survey
Source: J Foot Ankle Res. 2021 Nov 29;14:58. doi: 10.1186/s13047-021-00495-3 (PMC8628050; doi:10.1186/s13047-021-00495-3)
Supplement: Supplementary file 2 — Additional file 2. Full polychoric correlation matrix of variables; job role, footwear worn and lower limb problem. [file 13047_2021_495_MOESM2_ESM.docx]

Supplementary table 2: Polychoric correlation matrix, red highlighting indicates greater than 0.5
